# Scaling up a brief alcohol intervention to prevent HIV infection in Vietnam: a cluster randomized, implementation trial

**DOI:** 10.1186/s13012-024-01368-6

**Published:** 2024-06-12

**Authors:** Sophia M. Bartels, Huong T. T. Phan, Heidi E. Hutton, Do T. Nhan, Teerada Sripaipan, Jane S. Chen, Sarah L. Rossi, Olivia Ferguson, Ha T. T. Nong, Ngan T. K. Nguyen, Le Minh Giang, Hao T. M. Bui, Geetanjali Chander, Hojoon Sohn, Sol Kim, Ha V. Tran, Minh X. Nguyen, Byron J. Powell, Brian W. Pence, William C. Miller, Vivian F. Go

**Affiliations:** 1 Department of Health Behavior, The University of North Carolina Gillings School of Global Public Health, Chapel Hill, NC USA; 2 Vietnam Administration of HIV/AIDS Control, Hanoi, Vietnam; 3 Johns Hopkins Hospital University School of Medicine, Baltimore, MD USA; 4 UNC Vietnam, Hanoi, Vietnam; 5 Department of Epidemiology, Hanoi Medical University, Hanoi, Vietnam; 6 Division of General Internal Medicine, University of Washington School of Medicine, Seattle, WA USA; 7 Seoul National University College, Seoul, Korea; 8 Brown School, Washington University in St. Louis, St. Louis, MI USA; 9 Department of Epidemiology, The University of North Carolina Gillings School of Global Public Health, Chapel Hill, NC USA

**Keywords:** Implementation science, Experiential brief alcohol intervention, HIV, Vietnam

## Abstract

**Background:**

Evidence-based interventions (EBIs) often address normative behaviors. If a behavior is also common among clinicians, they may be skeptical about the necessity or effectiveness of an EBI. Alternatively, clinicians’ attitudes and behaviors may be misaligned, or they may lack the knowledge and self-efficacy to deliver the EBI. Several EBIs address unhealthy alcohol use, a common and often culturally acceptable behavior. But unhealthy alcohol use may be particularly harmful to people with HIV (PWH). Here, we present an implementation trial using an experiential implementation strategy to address clinicians’ knowledge, attitudes, and behaviors. Clinicians receive the experiential intervention before they begin delivering an evidence-based brief alcohol intervention (BAI) to PWH with unhealthy alcohol use.

**Methods:**

*Design*: In this hybrid type 3 implementation-effectiveness cluster randomized controlled trial, ART clinics (*n* = 30) will be randomized 1:1 to facilitation, a flexible strategy to address implementation barriers, or facilitation plus the experiential brief alcohol intervention (EBAI). In the EBAI arm, clinicians, irrespective of their alcohol use, will be offered the BAI as experiential learning. EBAI will address clinicians’ alcohol-related attitudes and behaviors and increase their knowledge and confidence to deliver the BAI.

*Participants*: ART clinic staff will be enrolled and assessed at pre-BAI training, post-BAI training, 3, 12, and 24 months. All PWH at the ART clinics who screen positive for unhealthy alcohol use will be offered the BAI. A subset of PWH (*n* = 810) will be enrolled and assessed at baseline, 3, and 12 months.

*Outcomes*: We will compare implementation outcomes (acceptability, fidelity, penetration, costs, and sustainability) and effectiveness outcomes (viral suppression and alcohol use) between the two arms. We will assess the impact of site-level characteristics on scaling-up the BAI. We will also evaluate how experiencing the BAI affected clinical staff’s alcohol use and clinic-level alcohol expectations in the EBAI arm.

**Discussion:**

This trial contributes to implementation science by testing a novel strategy to implement a behavior change intervention in a setting in which clinicians themselves may engage in the behavior. Experiential learning may be useful to address normative and difficult to change lifestyle behaviors that contribute to chronic diseases.

**Trial Registration:**

NCT06358885 (04/10/2024), https://clinicaltrials.gov/study/NCT06358885.

**Supplementary Information:**

The online version contains supplementary material available at 10.1186/s13012-024-01368-6.

Contributions to the literature

- This study tests a novel implementation strategy to educate ART providers and address their attitudes and knowledge about alcohol use and treatment in Vietnam. Few implementation strategies are grounded in an experiential approach to changing provider attitudes and behaviors in contexts where these behaviors are pervasive.
- It uses mixed-methods to determine mechanisms influencing scale-up of the brief alcohol intervention (BAI) to inform future implementation of alcohol interventions for people with HIV across settings where alcohol is normalized.
- It assesses resource-related costs associated with the experiential BAI (EBAI) and EBAI implementation, which can inform policymakers working to prevent HIV transmission.

## Background

Evidence-based interventions (EBIs) are designed to address numerous health behaviors that are to challenging to change because they are highly normalized and pervasive in society [1]. In these cases, the healthcare providers tasked with delivering the EBIs may also engage in or accept the normative behavior that these EBIs target. In such settings, providers may be less willing to adopt the EBI within their clinical practice, as they may perceive it to be ineffective or misaligned with their own attitudes and behaviors [2, 3]. Thus, providers’ knowledge, self-efficacy, attitudes, and behaviors may act as barriers to effective implementation and scale-up of EBIs [4–6].

One example of a behavior that is normalized in many settings is unhealthy alcohol use. Unhealthy alcohol use is defined as a spectrum of use from risky/hazardous to alcohol use disorder [7]. One group for which unhealthy alcohol use can have particularly harmful effects is people with HIV (PWH). Unhealthy alcohol use is common among PWH [8, 9], with 30% of PWH globally meeting at least one set of criteria for unhealthy alcohol use [8]. PWH with unhealthy alcohol use are less likely to adhere to antiretroviral therapy (ART) and be virally suppressed [9, 10], which may compromise treatment as prevention [11]. Brief alcohol interventions (BAIs) are proven effective for addressing unhealthy alcohol use among PWH [12, 13]; however, in settings where unhealthy alcohol use is normative, these EBIs may meet resistance from clinical staff (clinic staff members who interface with the clinical process).

To address clinical staff’s knowledge about and attitudes toward alcohol use and treatment, we have developed an experiential BAI (EBAI), a novel implementation strategy grounded in Experiential Learning Theory [6, 14]. Experiential interventions are an engaged learning process whereby individuals learn by doing and then reflect on the experience [14]. Experiential interventions have improved health behaviors, such as smoking and unhealthy diets [15, 16], among clients and counselors and changed providers’ attitudes towards patients with certain diseases [17]. For clinical staff, a direct experience with the BAI, combined with reflective observation of its effect, may lead to positive change in their attitudes and self-efficacy about the BAI delivery. This trial will be one of only a few studies that tests an experiential learning approach as an implementation strategy to increase providers’ knowledge about alcohol use and alcohol-related behaviors within a context where heavy alcohol use is pervasive and accepted throughout society.

As part of this cluster-randomized hybrid type 3 implementation-effectiveness trial, EBAI will be added to facilitation (FAC), an effective implementation strategy [18, 19]. In facilitation, facilitators with BAI expertise work with clinics to address implementation barriers, such as counselor skills and resource deficits [18, 19]. Facilitation alone will be the comparison condition. We hypothesize that EBAI plus facilitation will increase BAI fidelity at the clinic level and improve viral suppression in PWH with unhealthy alcohol use. Our aims are to:

1. Compare BAI implementation using facilitation only (FAC) to facilitation plus experiential BAI (FAC + EBAI) in ART clinics in Vietnam.
2. Explore the mechanisms of successful BAI scale-up in both the FAC and EBAI + FAC arms.
3. Measure the impact of EBAI on clinical staff.

In this protocol paper we describe the guiding theories and conceptual frameworks, study setting and design, randomization, and participants involved in this study. We also discuss the interventions, study outcomes, data collection, and analysis plan. We end this paper by describing this study’s innovations and potential challenges. We follow the Standards for Reporting Implementation Studies (StaRI) and CONSORT checklist in reporting the protocol for this study.

## Methods

### Guiding conceptual frameworks

This research is grounded in theories and conceptual frameworks to explain how EBAI incites change (Kolb’s Experiential Learning Theory [14]), the mechanisms through which EBAI operates (Aaron’s Role of Attitudes in Innovation Acceptance and Evidence-based Practice Implementation in Organizations Framework [6]), and how we can measure the impact of EBAI on BAI scale-up (Proctor’s Implementation Outcomes Framework [20]).

Kolb’s Experiential Learning Theory [14] underpins our experiential intervention. The theory posits that “learning is the process whereby knowledge is created through the transformation of experience.” It is represented by a four-stage learning cycle: 1) a concrete experience (e.g., EBAI), in which a new experience is encountered; 2) reflective observation, which provides the opportunity to process that experience into conceptual understanding of the intervention (e.g., EBAI consolidation components); 3) abstract conceptualization, whereby reflection gives rise to new ideas that the person has learned from their experience (e.g., EBAI consolidation components); and 4) active experimentation, where the learner applies the new concepts (e.g., delivery of BAI).

**Fig. 1:**
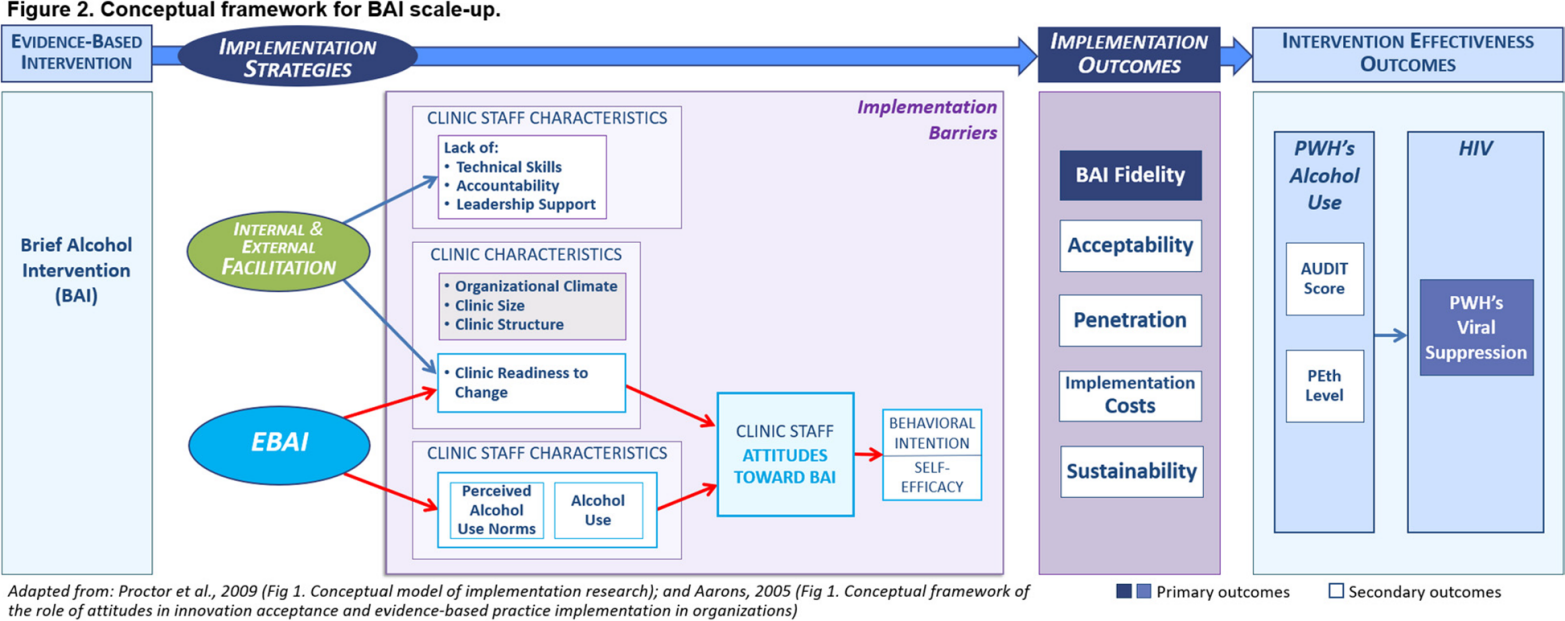
Conceptual model

Aarons’ framework posits that organizational and provider characteristics shape providers’ attitudes about an EBI, which influences if and how an EBI is implemented. For BAI scale-up, clinic readiness to change and clinical staff’s personal behaviors and perceived norms regarding alcohol use affect their attitudes towards BAI (Fig. 1). These attitudes affect their intention to deliver the BAI and their self-efficacy around delivering BAI; ultimately, staff’s acceptance of and fidelity to BAI is reduced.

We use Proctor’s Implementation Outcomes Framework [6, 21] to guide implementation outcome assessment, specifically acceptability, fidelity, penetration, cost, and sustainability. The effectiveness of BAI scale-up will be determined by the combination of facilitation and EBAI. We hypothesize that EBAI will improve BAI implementation (acceptability, fidelity, penetration, acceptability, fidelity, penetration, cost, and sustainability), which in turn will improve BAI effectiveness (alcohol use and viral suppression).

Our conceptual model (Fig. 1) nests our determinants framework (adapted from Aarons’ framework [6]) within our outcomes framework (adapted from Proctor’s Framework [20]). Moving from left to right, our conceptual framework illustrates how EBAI addresses attitudes towards BAI, which improves BAI fidelity, and in turn reduces PWH alcohol use and ultimately increases PWH viral suppression.

### Study setting

Similar to the global prevalence of unhealthy alcohol use, studies in Vietnam have found that 28% of PWH initiating or on ART have unhealthy alcohol use [1, 13]. One of the main barriers to reducing alcohol use among PWH in Vietnam is the normative view (particularly among men) that alcohol use is acceptable unless it causes social harms [1]. This tolerance of heavy alcohol use can lead to an ambiguous threshold between acceptable and harmful drinking. In formative research with staff at an ART clinic in Vietnam, we found that unhealthy drinking was pervasive among staff members: 74% of men, 17% of women, and 37% of clinical staff self-reported unhealthy drinking, based on the Alcohol Use Disorders Identification Test (AUDIT-C) criteria. Qualitatively, we found that staff were often expected to drink at work events, and they downplayed their drinking, often describing it as “occasional” despite unhealthy drinking scores, highlighting the lack of awareness and skewed self-perceptions of drinking.

### Study design

**Fig. 2:**
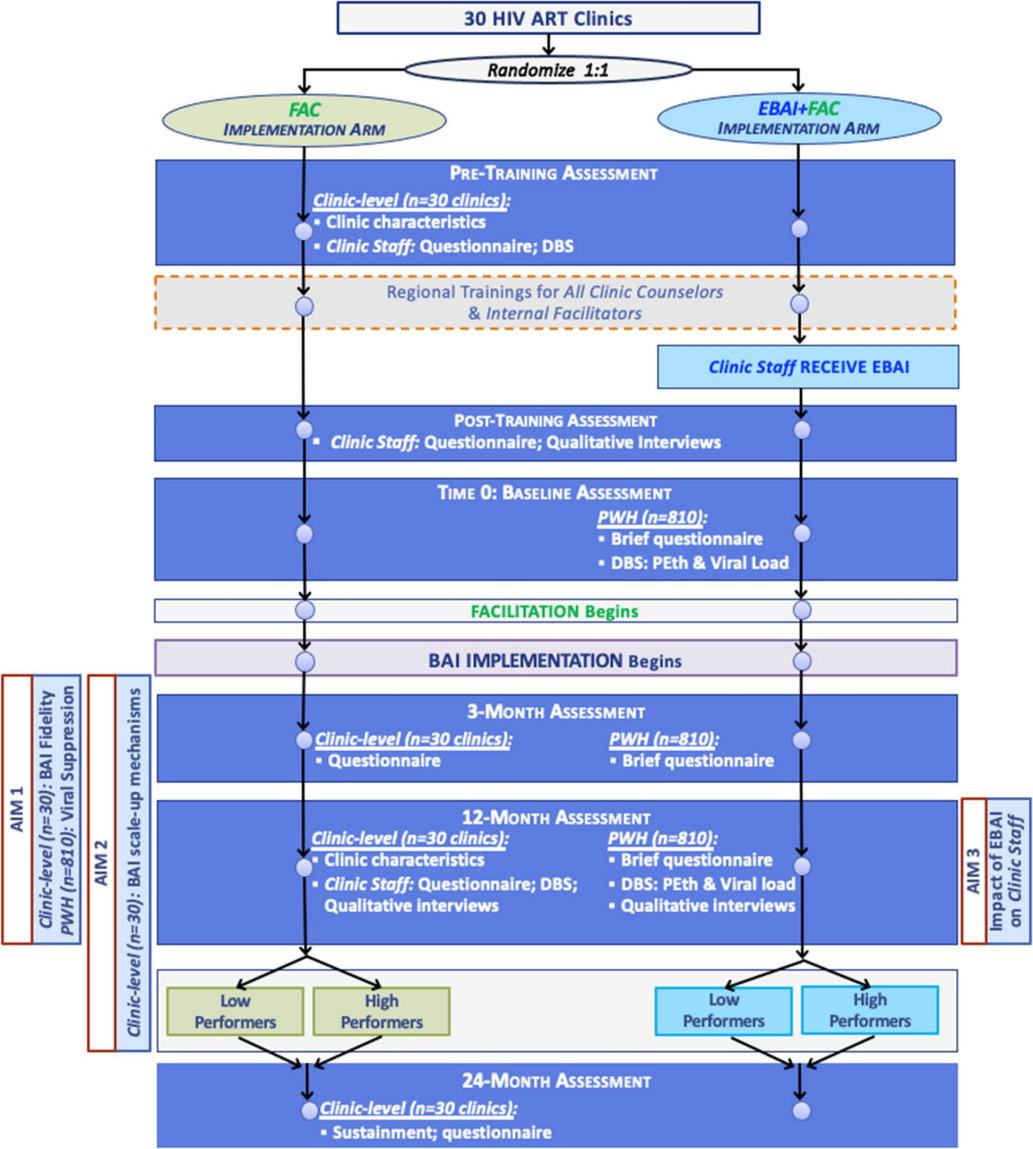
Study design

This study is a hybrid type 3, two-arm cluster-randomized implementation-effectiveness trial to assess the implementation of a BAI into ART clinics across Vietnam. ART clinics (*n* = 30) will be stratified by size and time of regional training, if feasible, to ensure comparability across arms, based on data from our initial site visits with each clinic. Implementation and effectiveness outcomes are assessed at the ART clinic level (*n* = 30) (Fig. 2).

### ART clinics

ART clinics will be selected with guidance by the Vietnam Administration of AIDS Control (VAAC), in 10 provinces (of 63) in 5 regions (of 6) in Vietnam. The clinics serve > 22,000 PWH on ART; each clinic has > 200 PWH on ART. The clinics were selected to reflect a range of sizes and urbanicity/rurality.

### Randomization

Randomization will be 1:1 with 15 clinics per arm. Random allocation will be implemented by study statisticians, who will use a random number generator to assign each clinic to an arm at a single point in time. Due to the nature of the intervention, clinics will not be masked, but data managers, statistical analysts, and staff who collect and/or manage outcome data will be masked to randomization results.

### Participants

#### PWH participants

All PWH attending the study clinics for ART, whether newly initiating or on ART, will be screened for the BAI intervention, using the AUDIT-C, as a part of new routine clinic procedure. PWH with an AUDIT-C score ≥ 4 for men or ≥ 3 for women and ≥ 18 years of age will be offered the BAI. PWH meeting eligibility criteria for the BAI will be consented to receive the BAI and to allow audio recording of their BAI sessions. It is estimated that around one third of all PWH on ART (up to 8000 PWH) at the study clinics will be eligible and agree to take part in the study and will be offered BAI. PWH receiving the BAI and not enrolled in the cohort described below will only provide data for the fidelity assessment of the counselors’ performance of the BAI sessions. A subset of PWH in each clinic, meeting BAI eligibility criteria, will be enrolled in the cohort to assess viral suppression and ART adherence (27 PWH/clinic (total *n* = 810)). Cohort eligibility criteria includes: (1) living with HIV; (2) currently attending a study ART clinic; (3) having an AUDIT-C score ≥ 4 for men or ≥ 3 for women; 4) ≥ 18 years of age; and 5) willingness to provide informed consent.

Recruitment of PWH participants will follow routine post-test counseling procedures or regular ART clinic visits. Those PWH meeting alcohol use eligibility will be given a written consent form for participation in the BAI and BAI session audio recording (for fidelity purposes, described below). PWH who do not screen positive for unhealthy alcohol use will continue to be screened with the AUDIT-C at future ART clinic visits. Each clinic’s PWH cohort sample will be distributed over months 2–10 of implementation. Each month, PWH will be recruited on consecutive days, or as necessitated by the clinic schedule, until 3 PWH are enrolled.

For the PWH cohort, participants will complete study assessments at baseline, 3-, and 12-months, including HIV history and ART use, AUDIT [22], mental health questionnaires [23, 24], and health-related quality of life (HRQoL), using the EQ-5D-5 L instrument [23–25] (among others). Dried blood spots (DBS) for phosphatidylethanol (PEth), a biomarker for recent alcohol use [26], and viral load testing will be collected at enrollment and 12 months. A subset of participants will participate in in-depth interviews at 12 months.

### ART clinic directors and staff

Eligibility criteria for ART clinic directors and staff include working at one of the study ART clinics as a clinic director, physician, nurse, or counselor and willingness to provide informed consent. All staff at the study ART clinics will be recruited to participate in the study (*n* = 120, ~ 4/clinic).

Clinical staff will complete assessments at pre-training, post-training, 3, 12, and 24-months. Surveys will assess clinic-level implementation factors, alcohol use and attitudes [22, 27–29], and acceptability, appropriateness, feasibility [30, 31], and sustainability of the BAI [32] (among others). Dried blood spots (DBS) for PEth will be collected at pre-training and 12-months.

### Interventions

#### BAI

BAI was adapted from the Healthy Women Study, an intervention for alcohol use among women with HIV in the US [12]. The Healthy Women Study was based on Project TrEAT, an intervention that was effective in men and women in diverse contexts [33]. In 2016, BAI was further adapted for use in Vietnam as part of REDART, a 3-arm randomized controlled trial (RCT) among 440 adults with unhealthy alcohol use receiving ART in 7 HIV outpatient clinics in Thai Nguyen, Vietnam [1, 13, 34]. PWH starting or on ART with AUDIT-C scores of ≥ 4 for men or ≥ 3 for women were randomly assigned to standard of care, BAI, or a combined intervention of motivational enhancement therapy and cognitive behavioral therapy. The BAI and combined intervention arms significantly reduced alcohol use compared to the standard of care. The BAI arm also increased viral suppression compared to the standard of care and was cost-effective [35].

During the current study, all eligible PWH participants at the study clinics, regardless of arm, will receive the BAI. BAI comprises 2 in-person sessions and 2 booster telephone sessions. Face-to-face 45-minute sessions occur ~ 1 month apart; 10-minute telephone sessions occur 2 to 3 weeks after each face-to-face session. The BAI’s core components, the critical elements that are needed for BAI to be effective, are listed in Table 1.

**Table 1:** BAI Core Components

**Session 1**
Introduction, intervention overview, purpose
Reasons for drinking
Consequences of alcohol use
What is a standard drink, lower risk versus unhealthy use
Personalized feedback on level of drinking
Reasons to cut down or quit
Triggers, cravings; Identifying risky moods and situations
Ways to manage risky moods and situations for self-efficacy
Optional goal settings for lower risk use or abstinence; change plan
Homework assignment: track alcohol use, triggers, cravings
**Session 2**
Review of events since last session: alcohol use patterns, triggers or cravings encountered; enactment of coping skills & outcome
Homework review: reasons to reduce; risky situations; how to handle risk
Tailored action plan: including
a) Engaging supportive others
b) Plans to meet drinking goals; facilitators and challenges
c) Coping skills/activities that do not involve alcohol
d) Refusal skills
e) Home and emergency strategies
Summary of personal action plan; Closing/follow up

### Implementation strategy development through implementation mapping

Implementation Mapping is a 5-step process that incorporates theory, evidence, and stakeholder perspectives to ensure that implementation strategies reflect stakeholder preferences and clarify the mechanisms through which strategies exert their effects. During the first 3 months of this project, we engaged leaders from the VAAC and provincial CDCs in the Implementation Mapping process. In steps 1 and 2, we conducted an implementation needs assessment and confirmed outcomes, performance objectives, and determinants. In step 3, we selected implementation strategies to address identified determinants of change. We proposed core strategies in both arms, based on our preliminary studies. We refined these strategies using Implementation Mapping to clearly operationalize each study condition and associated strategies (see Additional File 1). We developed matrices that link performance objectives, determinants, implementation strategies and behavior change methods, and implementation outcomes. In step 4, we developed implementation protocols and materials, and in step 5, we will evaluate the implementation outcomes.

### Control arm: FAC

In many settings, passive, low-level support, such as manuals and training, is inadequate to implement HIV-related EBIs [36]. Facilitation is a flexible, effective implementation strategy in which a facilitator with expertise in the EBI works with providers to address common barriers to EBI implementation and scale-up. Facilitation is commonly used to implement new programs [18, 19] and has a strong evidence base [18, 19, 37, 38]. In the trial, we will include both external facilitation (someone outside the clinics provides technical expertise in adopting the EBI) and internal facilitation (someone within the clinic helps align the EBI with clinic values and priorities). External facilitators (from the central team) will work with internal facilitators, who clinic leadership will identify with input from clinical staff, to carry out implementation strategies selected using Implementation Mapping. Internal facilitators will help identify challenges to implementation within the clinic and work with the external facilitators to identify solutions. Facilitation will happen at regular intervals as part of regional calls.

### Intervention arm: EBAI + FAC

While facilitation’s widespread acceptance supports its use for BAI scale-up, facilitation alone may not be enough. EBAI + FAC combines an experiential intervention with facilitation. In the 15 EBAI + FAC clinics, clinical staff (~ 4/clinic) will be offered the BAI, regardless of their self-reported alcohol use. We will include all staff, not just counselors, to facilitate changing clinic-level alcohol expectations, to reduce the potential for confidentiality breaches, and to give all staff experience with the BAI. After consent, a centralized counseling team will deliver the BAI to clinical staff. If a staff member is uncomfortable discussing their own alcohol use or reports minimal alcohol use, they may choose to adopt the persona of someone with unhealthy alcohol use and experience the BAI through role-play.

After receiving EBAI, EBAI + FAC counselors will participate in 3 consolidation components which will include 2 central counselor-led sessions and a reflection exercise. The first session will be an integration session to help counselors integrate and process their experiences. The reflection exercise will allow counselors to reflect on how receiving the intervention may have changed how they delivered the intervention to PWH. The second session will be a brief check-in to see how they are doing after the EBAI experience and delivering the BAI to PWH.

### Outcomes

#### Implementation outcomes

The primary implementation outcome will be fidelity to the BAI as delivered to PWH participants. Fidelity will be measured at the clinic level at 15 months using a clinic fidelity score (12 months of enrollment with an extra 3 months for BAI completion). The score comprises successful completion of the 4 protocol-specified sessions (2 in-person, 2 phone) within 7 weeks of the initial session weighted by the central fidelity rater’s quality rating of the in-person sessions. Session completion will be assessed by reviewing counselor logs; session quality will be assessed by an expert rater through review and scoring of up to 15% of counseling session audio-recordings. The primary outcome, fidelity, will be assessed using a tailored selection of fidelity measures including the BAI Core Components Checklist. The clinic fidelity score ranges from 0 to 100 (higher scores indicate higher fidelity). The score will be the percentage of counseling sessions completed, multiplied (weighted) by the combined average quality rating of counseling sessions. Fidelity = % BAI sessions completed * average BAI session quality rating. Raters will be masked to the study arm when reviewing audio-recordings.

Secondary implementation outcomes include acceptability, penetration, implementation costs, sustainability, and fidelity with a 4-month completion window (Table 2).

**Table 2:** Primary and Secondary Study Outcomes

Outcome	Definition	Measure or scale
*Primary implementation outcome*		
Fidelity to BAI as delivered	Delivering BAI as intended (within 7 weeks of the initial session)	% BAI sessions completed × average quality score
*Secondary implementation outcomes*		
Acceptability of BAI	Perception that BAI is agreeable, palatable, or satisfactory	*For BAI counselors*: Mental Health Implementation Science Tools (mhIST) Acceptability Scale for providers [31] *For clinic staff*: Acceptability of Intervention Measure (AIM) [30] *For PWH participants*: Mental Health Implementation Science Tools (mhIST) Acceptability Scale for consumers [31]
Penetration	(1) Proportion of PWH initiating or on ART who are screened with the AUDIT-C; (2) Proportion of PWH that screen positive receiving at least one counseling session	Screening log: AUDIT-C & 1st session
Cost	Costs of BAI and its implementation	Directly measured non-research costs, including all costs of implementation
Sustainability	% of clinics continuing to offer BAI after completion of the study	Provider support of sustainment scale (PRESS) [32]
Fidelity [extended window]	Delivering BAI as intended (within 4 months of the initial session)	% BAI sessions completed × average quality score
*Primary effectiveness outcome*		
Viral Suppression	% participants virally suppressed	viral load < 1000 copies/mL on a DBS sample
*Secondary effectiveness outcomes*		
Clinical staff alcohol use	Heavy drinking days, number of drinking days, and number of drinks per drinking day	AUDIT
PWH alcohol use	Heavy drinking days, number of drinking days, and number of drinks per drinking day	AUDIT

#### Effectiveness outcomes

The primary effectiveness outcome is viral suppression (< 1000 copies/mL) at 12 months among the cohort sample. Viral suppression is defined as an undetectable viral load on a DBS sample. Secondary outcomes will be AUDIT (total score) [22] and PEth level among the cohort sample. PEth will be analyzed dichotomously (using a cut-off of > 20 ng/ml for unhealthy alcohol use) and continuously [39, 40].

#### Cost and cost-effectiveness outcomes

Economic evaluation will be structured in 3 assessments of costs and cost-effectiveness of BAI and EBAI. First, we will assess implementation and BAI intervention delivery costs in the respective study arms. Second, we will assess the cost-effectiveness of the EBAI + FAC relative to FAC based on incremental costs of implementation weighed against the differences in the implementation and effectiveness outcomes. Third, we will assess differences in patient perspective costs resulting from participating in the respective BAI intervention.

Using the implementation costing framework [41], we will assess total cumulative implementation costs and service delivery unit costs of EBAI + FAC and FAC and assess incremental costs of the intervention arm relative to the control. Implementation costs will be further assessed for individual sites and by key implementation activities (see Additional File 2). We will then explore study sites’ operational factors, measures of implementation outcomes, and implementation activity time estimates to evaluate factors contributing to incremental implementation costs and variabilities in costs. Cost-effectiveness will be assessed based on the incremental cost-effectiveness ratio (ICER) estimate, which will be calculated from (1) a comparison of total implementation costs and fidelity scores, (2) incremental service delivery costs of EBAI + FAC compared against changes in viral suppression and estimated Quality Adjusted Life Years (QALYs) gained through the EBAI + FAC relative to FAC. QALYs will be estimated using a Markov model of the population eligible for EBAI + FAC or FAC, constructed based on viral suppression, ART adherence, alcohol use, other patient-level factors from our study and literature estimates.

Patient costs and empiric quality of life estimates will also be assessed. Patient costs include data on socio-economic status, costs associated with alcohol use, and direct and indirect costs associated with HIV and alcohol disorder-related care. Patient costs will be calculated for each follow-up period and assessed as cumulative per patient costs during the study’s follow-up period. We will also evaluate the effect of the BAI intervention on participants’ HRQoL measured using the EQ-5D-5 L. We will perform uni- and multivariate linear regression (with total cumulative patient costs as the outcome measure) to examine the association between patients’ socio-economic, epidemiologic, and clinical factors influencing patient perspective costs and HRQoL.

#### High- and low-performing sites

Using a triangulation (QUAN + QUAL) mixed methods design [42], we will examine factors that influence successful scale-up, guided by our conceptual framework. We will classify clinics in both arms as high- or low-performing, based on clinic-level BAI fidelity and viral suppression. Our a priori definition of successful implementation at 12 months is a fidelity score of 60 and 85% viral suppression among PWH who received the BAI. Fidelity score and viral suppression cut-offs are based on the Vietnam sites’ experiences in REDART (92% viral suppression in the BAI arm; 77% in the standard of care arm).

As part of this mixed methods analysis, we will explore relationships with clinic characteristics (Table 3) (e.g., organizational climate [31], readiness to change [43], implementation leadership [44], implementation climate [45]), clinical staff characteristics (e.g., skills, alcohol norms [27]), level of engagement with facilitation, and, in EBAI + FAC arm, EBAI uptake and impact. This data will come from surveys with clinical staff and PWH conducted throughout the study and in-depth interviews conducted at 12-months with a sub-sample of clinical staff and PWH.

**Table 3:** Additional measures

**Construct**	**Measure**	**Schedule**
Clinical Staff Measures^a^		
Demographics	Standardized questionnaire	PreT
Alcohol use biomarker	PEth	PreT, 12 mos
Alcohol use	AUDIT [22]	PreT, PoT, 3, 12, 24 mos
Alcohol norms	Drinking Norms Scale [27]	PreT, PoT, 3, 12, 24 mos
Alcohol attitudes	Short Alcohol and Alcohol Problems Perception Questionnaire [28]	PreT, PoT, 3, 12, 24 mos
Alcohol abstinence stigma	Alcohol abstinence stigma scale [29]	PreT, PoT, 3, 12, 24 mos
Mental health	PHQ-2	PreT, PoT, 12, 24 mos
BAI appropriateness	Counselors: mhIST [31], provider version All other clinic staff: IAM [30]	12 mos
BAI feasibility	Counselors: mhIST [31], provider version All other clinic staff: FIM [30]	12 mos
EBAI acceptability	mhIST [31], consumer version	12 mos
EBAI feasibility	Developed for study	12 mos
Readiness for change	Organizational Readiness for Implementing Change [43]	PreT, PoT, 12, 24 mos
Implementation leadership	Implementation Leadership Scale (staff and supervisor versions) [44]	PreT, PoT, 12, 24 mos
Implementation climate	Jacobs et al., 2014 [45]	3, 12, 24 mos
Organizational climate	mhIST [31], provider version	3, 12, 24 mos
Organizational measures	Theoretical Domains Framework [46]	3, 12, 24 mos
PWH Participant Measures^a^		
Demographics	Standardized questionnaire	0, 3, 12 mos
Alcohol use biomarker	Peth, DBS	0, 12 mos
Alcohol use	AUDIT [22]	0, 3, 12 mos
Alcohol use reasons	Standardized questionnaire	0, 3, 12 mos
Alcohol abstinence stigma	Alcohol abstinence stigma scale [29]	0, 3, 12 mos
HIV and ART	Standardized questionnaire	0, 3, 12 mos
HIV viral load	DBS	0, 12 mos
BAI appropriateness	mhIST [31], patient version	3 mos
Mental health	Patient Health Questionnaire-8 [23], Generalized Anxiety Disorders-7 [24]	0, 3, 12 mos
Cost, direct & indirect	Standardized questionnaire	0, 12 mos
Health-related quality of life	EQ-5D [25]	0, 3, 12 mos
Working alliance	Working Alliance Inventory [47]	3 mos
Drug & injection drug use	Standardized questionnaire	0, 3, 12 mos

^a^Note: this is not necessarily an exhaustive list of the measures that will be used

#### Impact on clinical staff

We will use mixed methods triangulation (QUAN + QUAL) [42] to assess the effects of BAI delivery and EBAI receipt on clinical staff’s alcohol use and expectations. These data will come from clinical staff surveys conducted throughout the study and from in-depth interviews conducted at 12 months with a sub-sample of clinical staff. The primary outcome will be change in AUDIT score from baseline to 12 months. We hypothesize that clinical staff in EBAI + FAC arm will reduce their own alcohol use and report reduced clinic-level alcohol use expectations more than clinical staff in the FAC arm.

### Data collection and analysis

#### Formative data collection and BAI adaptation

We are in Year 1 of this study and plan to start enrollment in 2024. Prior to randomization and baseline assessment, we used Implementation Mapping to ensure that the implementation strategies reflected stakeholder preferences and to clarify the mechanisms through which the strategies exert their effects.

Concurrently with the Implementation Mapping process, we adapted the BAI manual for delivery to clinical staff and for scale-up across Vietnam. The first phase of adaptation included holding discussions with local HIV organizations about adaptations to the manual. The second phase included administering the manual to ART clinical staff and conducting IDIs with clinical staff (across three clinics not participating in the RCT) to further adapt it.

Next, we conducted cognitive interviewing of pictures for the manual and some of the patient assessment scales. We administered these cognitive interviews to around 20 PWH with unhealthy alcohol use across the same 3 ART clinics selected for the adaptation IDIs. After this cognitive interviewing, we pilot tested PWH sub-cohort assessment measures with around 5 additional PWH at one of the clinics. We then pilot tested the BAI with ~ 5 counselors, with the goal of testing and further adapting the materials from the experiential learning process.

During the pre-implementation phase, we also conducted site initial assessments and site in-person visits. The survey was sent to sites to explore current alcohol screening and treatment services provided, current alcohol treatment referral protocol, estimated number of patients with unhealthy alcohol use, and other relevant characteristics.

#### Trial data

*Quantitative data.* For Aim 1, the comparison of FAC to EBAI + FAC, survey data will be collected from clinical staff (at pre-training, post-training, 3, 12, and 24 months) and PWH participants in the sub-sample cohort (at baseline, 3, and 12 months). Viral suppression and PEth will also be collected for PWH participants in the sub-sample cohort using the DBS. From clinic records, we will also collect data at the clinic level on clinic demographics, clinic viral suppression, number of patients, number of patients screened and number with a positive AUDIT score, and number who accept and receive the BAI.

We will conduct intention-to-treat comparisons between arms. Fidelity will be compared using a t-test. We will also conduct a sensitivity analysis of the fidelity outcome that uses a broader window for completion. Additional analyses will use multiple linear regression to adjust for variables likely to be associated with the outcome, defined a priori. Penetration will be compared using generalized estimating equations with a logit link function and binomial error distribution; additional variables included in the two penetration models will be defined a priori. Viral suppression will be analyzed using generalized estimating equations with a logit link function and binomial error distribution, accounting for clustering within clinic. Additional staff and PWH-level outcomes will be analyzed with similar approaches.

For Aim 2, site characteristics will be assessed with exploratory analyses to examine associations with high- and low-performing clinics. We will use a generalized linear model with a logit link and binomial error distribution to assess the dichotomous outcome of high or low performance. Each clinic characteristic, clinical staff measure, facility engagement measure, and EBAI-related measure (Table 3) will be included in separate models as a single explanatory variable with an indicator variable for study arm and an interaction term (α = 0.10). This approach allows us to examine the impact of each factor on EBAI + FAC or FAC alone. Two sets of models will be used: baseline measures only and change from baseline to evaluation (for measures assessed over time).

For Aim 3, at pre-training, post-training, 3, 12, and 24 months, consenting clinical staff will complete the AUDIT and Alcohol Abstinence Scale; DBS will be collected for PEth at pre-training and 12 months. The proportion of clinical staff receiving and completing the BAI in EBAI + FAC arm will be assessed. The primary analyses will compare staff in the two arms using generalized estimating equations with an identity link and Gaussian error distribution to compare means (AUDIT scores). Secondary analyses will address individual elements of the AUDIT (e.g., heavy drinking days, drinks per drinking day) and alcohol abstinence stigma. Because self-reported data may be subject to social desirability bias, we will also use PEth examined both as a dichotomous variable (> 20 ng/ml) and continuously [39, 40]. Generalized estimating equations with appropriate link function and error distributions will be used for secondary analyses.

*Qualitative data.* As part of Aim 2, after regional training and EBAI, we will conduct in-depth interviews with BAI counselors (*n* = 16) and other staff (*n* = 16) to understand attitudes toward BAI (total *n* = 32) in 4 clinics from each arm. We will ask participants about experiences with alcohol use before and after receiving the training, perceived norms around alcohol use, and attitudes toward BAI for PWH on ART.

We will also conduct 4 semi-structured interviews with PWH who completed the BAI ≥ 6 months earlier and with BAI counselors (*n* = 16) and other clinical staff (*n* = 16) in each of 2 sites of the following 4 site performance types (8 clinics; 32 interviews over 2 years): (1) EBAI + FAC low performers; (2) EBAI + FAC high performers; (3) FAC low performers; and (4) FAC high performers. These interviews will inform the context and processes that may underline successful scale-up of BAI including the mechanisms of change for the implementation strategies and refinements that may be needed for future scale-up efforts.

All qualitative interviews will be audiotaped, transcribed, translated, and coded using NVivo software. Analysis will begin as data are collected so that topics for further exploration can be incorporated into ongoing fieldwork. Textual data analysis will involve: (1) reading for content; (2) deductive and inductive coding; (3) data display to identify emerging themes; (4) data reduction; and (5) interpretation. Clinic staff responses will be compared within and across the staff groups, gender, and within and across high- and low-performing sites. Interview and quantitative data are being used for convergence and will be merged and triangulated to understand mechanisms and pathways underlying clinic implementation performance.

For Aim 3, during the staff interviews conducted for Aim 2, we will ask questions related to the staff members’ personal alcohol use and general attitudes related to alcohol use within the clinic. Among staff in EBAI + FAC arm, the interviews will explore how receiving EBAI influenced their alcohol use and attitudes towards BAI more generally.

#### Economic evaluation data

Cost data related to implementation process and service delivery will be measured using budgetary analysis, time assessment studies, implementation costing tools, and a review of study management logs/records. Resources and cost data will be categorically assessed based on key resource types from the health service provider perspective and the activity-based costing approach, ascertaining resource use and costs based on implementation and service delivery discrete activities defined for our study. All cost data will be mapped for discrete implementation and service delivery activities and reviewed on a periodic basis. Patient costs will be collected for all cohort study participants using a modified version of the patient cost survey questionnaire developed from our earlier studies [35, 48, 49]; patients selected for clinical evaluation will be assessed for their HRQoL at follow-up assessments.

### Sample size

#### Implementation outcomes sample size calculations

For the primary outcome of fidelity, using two-tailed tests and α = 0.05 and assuming a conservatively large standard deviation of 15, 30 sites will give us 80% power to detect a difference between a fidelity score of 68 in EBAI + FAC arm and 52 in the FAC arm (corresponds to 72% session completion and 72% average quality ratings).

#### Effectiveness outcome sample size calculations

We estimate that the intraclass correlation may range from 0.01 to 0.05, as is typical in clinic-level behavioral interventions, implying a design effect between 2.5 and 8.4. Assuming the same intraclass correlation of 0.01–0.05, with a sample of *n* = 720 (~ 24 per clinic after accounting for deaths), we will have ≥ 80% power to detect differences of 10–14% points in viral suppression between arms (e.g., 85% vs. 75% if intraclass correlation = 0.01; 85% vs. 72% if intraclass correlation = 0.05).

## Discussion

Implementation strategies designed to increase EBI uptake by clinicians less commonly address their attitudes toward the EBI or the behavior that they are trying to change in settings in which the behavior is widely accepted and/or pervasive. For normative behaviors in a given socio-cultural setting, such as alcohol use, provider and counselor perspectives and own experiences can represent a critical barrier to intervention implementation [50–52]. In this trial, we will offer clinical staff an opportunity to experience the intervention firsthand, directly addressing attitudes about the BAI itself and alcohol norms more broadly. This novel approach to implementation has direct implications for clinician-focused interventions related to alcohol use and many other conditions.

Based on Experiential Learning Theory, clinical staff will undergo the BAI themselves and reflect on the experience to gain self-efficacy for BAI delivery. This approach may affect changes in staff’s attitudes about alcohol use and reduce staff’s own alcohol consumption. This experiential approach may encourage staff buy-in to the importance and effectiveness of the BAI for PWH.

This study addresses implementation science priorities in several ways:

- It tests an innovative implementation strategy, experiential learning, which if found to be successful at altering clinicians’ attitudes, could hold promise as an implementation strategy for changing clinician attitudes in other settings where heavy alcohol use is normalized or for other interventions that aim to reduce commonly accepted behaviors. It also moves beyond testing experiential learning versus a control condition, as we have already established that facilitation has a strong evidence-base as standard of care for intervention scale-up [18, 19, 37, 38].
- It utilizes implementation mapping to create a “baseline” package of implementation strategies that will be implemented across both study arms to better address contextual barriers to BAI implementation [53]. There is a need in implementation science to more systematically select implementation strategies that are most likely to lead to change and close the research-to-practice gap [54, 55].
- It assesses the costs and cost drivers of the FAC and EBAI implementation process and BAI service delivery. Furthermore, we ascertain patient perspective costs measured longitudinally. These data will be essential for helping policymakers in their decision-making around using EBAI as a cost-effective implementation strategy. Given the lack of studies systematically evaluating costs and cost-effectiveness of implementation and implementation studies, our study addresses an important evidence gap and provides methodological overview of conducting economic evaluation in implementation science trials [54, 56–59].
- Identifying potential mechanisms of implementation strategies, with a focus on how context impacts implementation success [60], is a high priority for implementation science [54, 61]. As part of this study, will examine the contextual factors, mechanisms, and pathways that influence successful BAI scale-up by identifying high- and low- performing clinics in both study arms.

While this study is grounded in a number of the priority areas in implementation science, one challenge is the logistics of implementing a trial in 10 provinces. This challenge is mitigated by our well-trained and experienced study team and implementing partners who help oversee implementation and provide technical assistance. A second concern is that staff in EBAI + FAC arm may be reluctant to experience the BAI as themselves because they might not be ready to disclose their unhealthy alcohol use. To help address this concern, clinical staff may opt to experience the BAI using role play or decline participation altogether. We will also intentionally use a distinct central team, unrelated to the clinic, to deliver EBAI to protect confidentiality.

## Conclusions

In settings with heavy alcohol use, alcohol reduction interventions are needed to improve ART adherence among PWH with unhealthy alcohol use. For successful scale-up of alcohol interventions in contexts where alcohol use is normative, strategies may be needed to address clinical staff’s attitudes toward alcohol use and alcohol treatment. This trial provides a novel approach to overcome these attitudes: experiential BAI delivery to clinical staff prior to its use for PWH. This trial will guide policymakers worldwide who are charged with preventing HIV transmission. Regardless of the trial outcome, this guidance will accrue based on the trial’s use of facilitation, implementation outcome results, effectiveness outcomes, costing, and characterization of the mechanisms of successful BAI scale-up.

### Supplementary Information

Additional file 1: Characteristics of standard implementation strategies. Description of standard implementation strategies from implementation mapping.

Additional file 2: Key implementation activities list. List of the key implementation activities for costing assessment.

## Data Availability

Data collection for this study is ongoing, so no data and materials are currently available.

## Abbreviations

ART: Antiretroviral therapy
BAI: Brief alcohol intervention
CDC: Centers for Disease Control
DBS: Dried blood spot
EBAI: Experiential brief alcohol intervention
EBI: Evidence-based intervention
FAC: Facilitation
HRQoL: Health-related quality of life
HIV: Human immunodeficiency virus
ICER: Incremental cost-effectiveness ratio
PEth: Phosphatidylethanol
PWH: People living with HIV
QALY: Quality Adjusted Life Years
RCT: Randomized controlled trial
UNC: University of North Carolina
VAAC: Vietnam Authority of AIDS Control
