# Supplementary material for: Scaling up a brief alcohol intervention to prevent HIV infection in Vietnam: a cluster randomized, implementation trial
Source: Implement Sci. 2024 Jun 12;19:40. doi: 10.1186/s13012-024-01368-6 (PMC11170841; doi:10.1186/s13012-024-01368-6)
Supplement: Supplementary file 1 — Additional file 1: Characteristics of standard implementation strategies. Description of standard implementation strategies from implementation mapping. [file 13012_2024_1368_MOESM1_ESM.pdf]

Additional File 1: Characteristics of standard implementation strategies

Implementation strategies	Actors	Action	Action Target	Dose	Timing
-Central team introduces BAI to VAAC<br>1. Review the BAI materials, associated implementation strategies, and highlight advantages of BAI over what is currently done<br>2. Meeting with VAAC to discuss implementation science and Implementation Mapping<br><br>-Build relationships and engagement*<br>*This is essential across all VAAC activities	National (VAAC) leaders	Central team prepares resources and organizes a meeting with VAAC to introduce the BAI, implementation science, and discuss collaboration	VAAC is the governmental organization in charge of HIV/AIDS care in Vietnam and also one of the main collaborators of the study. It is critical that VAAC understands the BAI intervention, the resources needed for BAI implementation at different levels, and has a basic understanding of implementation science so that they can provide support for BAI implementation at the national level	Once	~1 year before BAI implementation
Conduct initial site visits (with support from Central Team) to:<br>1. Build rapport with clinical staff and clinics' board of directors<br>2. Clarify staffing and infrastructure requirements for delivery of the BAI intervention		• Design a site assessment tool and send it to sites to be completed (done by Central team)<br>• Complete the assessment and return to central team for analysis (done by site staff)<br>• Visit sites participating in the study to build the initial rapport with members of the board of directors and site staff<br>• Introduce BAI and discuss the plan for collaboration with the sites to implement BAI<br>• Explore current routines for alcohol use screening and treatment, evaluate sites' resources and willingness to implement BAI<br>• Identify the list of potential staff to participate in launching events, trainings, and BAI activities<br>• Identify appropriate site staff to participate in BAI and seek approval from site leaders (done by all actors)	• Data on site infrastructures and personnel is collected from sites to inform the feasibility of implementation strategies, to build rapport with clinical staff and clinics' board of directors, and to clarify staffing and infrastructure requirements for delivery of the BAI intervention<br>• In order to implement BAI well, site leaders and staff need to understand the nature of the BAI intervention, design of an implementation study, timeline, budget, human resources needed, and their roles and responsibilities<br>• Since leadership support is critical to successful implementation of BAI, provincial and site leaders should be involved in the planning of BAI and feel empowered<br>• Appropriate site staff is identified to participate in launching events, training events and to implement BAI	Once	~1 year before BAI implementation

1. Conduct visits to Provincial CDC leaders/Provincial Health Dept. to introduce the BAI<br>2. Identify focal points in Provincial CDC leaders/Provincial Health Dept.		VAAC and central team prepare resources and organize a meeting with Provincial CDC leaders/Provincial Health Dept. to introduce the BAI, discuss collaboration, and work with them to identify the focal points of contact for BAI implementation	In order to ensure effective implementation of BAI at the sites, it is important to identify focal points of contact at each Provincial CDC/Provincial Health Dept. They will help with coordinating the intervention at the provincial level.	Once	~1 year before BAI implementation
1. Issue central-level guidelines to the provinces to scale-up BAI<br>2. Conduct regional launching events and training with Provincial CDC leaders/Provincial Health Dept. and central team for sites<br>- Both these strategies will emphasize the alignment of the BAI with the Government's National Strategy for Prevention and Control of non-communicable diseases for the period 2015-2025 with relevant stakeholders and health staff to facilitate intervention scale-up		• VAAC drafts and issues central-level guidelines to provinces to scale-up the BAI<br>• Provide an overview of BAI intervention for local leaders<br>• Give speeches to show local leadership support and willingness to implement BAI (done by VAAC and local leaders)<br>• Conduct a training on the BAI intervention, knowledge and skills for BAI delivery, the data collection system and other research procedures (with central team and Provincial CDC leaders/Provincial Health Dept.)	• All stakeholders understand the nature of BAI intervention, design of an implementation study, timeline, budget, and human resources needed for the study at sites, so that they motivated to collaborate and implement BAI<br>• Leadership support and political will also motivate site staff to participate in BAI and implement BAI well<br>• Site staff understands BAI intervention and their responsibilities. They are also trained to have the necessary skills and knowledge to implement BAI well at their sites	Once	Before BAI implementation
-Draft and circulate the roll-out schedule<br>-Negotiate timeline for start-up with each site		Develop and share roll-out schedule with sites	Study sites need to know the timeline and implementation activities, as well as their responsibilities, so that they can collaborate well with VAAC and the central research team to implement BAI	Once	Before BAI implementation

VAAC introduces BAI to Provincial CDC leaders/Provincial Health Dept.<br>- Review the BAI materials, associated implementation strategies, and highlight advantages of BAI over what is currently done<br>- Build relationships and engagement*<br>*This is essential across all Provincial CDC leaders/Provincial Health Dept. activities	Provincial CDC leaders/ Provincial Health Dept.	VAAC and central team prepare resources and organize a meeting with Provincial CDC leaders/Provincial Health Dept. to introduce the BAI and discuss collaboration	It is critical that the Provincial CDC leaders/Provincial Health Dept. understand the BAI intervention and the resources needed for BAI implementation at different levels so that they can provide support for BAI implementation at the provincial level	Once	~1 year before BAI implementation
1. Issue a provincial-level directive to the clinic directors to implement BAI at their site<br>2. Conduct regional launching events and training with VAAC and central team<br>*Note: this is the same strategy as listed under VAAC, just repeated given that there are multiple actors<br>- Both these strategies will emphasize the alignment of the BAI with the Government's National Strategy for Prevention and Control of non-communicable diseases for the period 2015-2025 with relevant stakeholders and health staff to facilitate intervention scale-up		• Provincial CDC leaders/Provincial Health Dept. draft and issues provincial-level directive to clinic leaders to implement the BAI<br>• Provide an overview of BAI intervention for local leaders (with VAAC and central team)<br>• Give speeches to show local leadership support and willingness to implement BAI (done by VAAC and local leaders)<br>• Conduct a training on the BAI intervention, knowledge and skills for BAI delivery, the data collection system and other research procedures (with VAAC and central team)	• All stakeholders understand the nature of BAI intervention, design of an implementation study, timeline, budget, and human resources needed for the study at sites, so that they motivated to collaborate and implement BAI<br>• Leadership support and political will also motivate site staff to participate in BAI and implement BAI well<br>• Site staff understands BAI intervention and their responsibilities. They are also trained to have the necessary skills and knowledge to implement BAI well at their sites	Once	Before BAI implementation

1. Issue a clinic-level directive to the staff to implement BAI<br>2. Central team, VAAC, and Provincial CDC leaders/Provincial Health Dept. conduct regional launching event and training<br>- Both these strategies will emphasize the alignment of the BAI with the Government's National Strategy for Prevention and Control of non-communicable diseases for the period 2015-2025 with relevant stakeholders and health staff to facilitate intervention scale-up	Clinic director	• Clinic directors draft and issue clinic-level directive to site staff to implement the BAI<br>• Provide an overview of BAI intervention for local leaders<br>• Give speeches to show local leadership support and willingness to implement BAI (done by VAAC and local leaders)<br>• Conduct a training on the BAI intervention, knowledge and skills for BAI delivery, the data collection system and other research procedures (with VAAC and Provincial CDC leaders/Provincial Health Dept.)	• All stakeholders understand the nature of BAI intervention, design of an implementation study, timeline, budget, and human resources needed for the study at sites, so that they motivated to collaborate and implement BAI<br>• Leadership support and political will also motivate site staff to participate in BAI and implement BAI well<br>• Site staff understands BAI intervention and their responsibilities. They are also trained to have the necessary skills and knowledge to implement BAI well at their sites	Once	Before BAI implementation
Revise staff roles (Identify clinical staff (directors, counselors, physicians, nurses, etc.) who will participate in BAI and assign specific roles to them (internal facilitator, AUDIT screening, counseling, etc.))		• Clinic directors review current staff roles and responsibilities<br>• Assign new BAI-specific roles	• Site staff are trained and have the necessary skills and knowledge to implement SNaP well at their sites AND are not overburdened by care duties	On-going	Ongoing (before BAI implementation, and throughout the study, as needed)
Review and revise the procedures and flow of a routine clinic visit of HIV patients to incorporate AUDIT screening and BAI counseling for patients		• Clinic directors review current clinic procedures and determine how BAI activities can fit within them<br>• Revise clinic visit procedures and flow to include BAI activities and communicate to staff members about new procedures and flow	• BAI screening and delivery does not disrupt clinic flow or duplicate already existing processes	Once	Ongoing (before BAI implementation, and throughout the study, as needed)
Integrate plans to address turnover, such as replacing and re-training for new staff		• Inform the central research team of staff turnover and replacement (done by site leaders and staff)<br>• Conduct training for new staff	• The central research team needs to be properly informed of turnover events, so that they can provide training for new staff.<br>• It is important that new staff understands their responsibilities and has the knowledge and skills to deliver BAI as intended.	On-going	Ongoing (after turnover events)

Ensure availability of a private and comfortable office space		• Clinic directors review current office spaces<br>• Designate an office space at the clinic for BAI activities and ensure that it has the required materials/set-up for BAI counseling	• Participants feel comfortable in BAI intervention office<br>• BAI counselors are able to deliver the BAI to every eligible patient with fidelity and in a private and comfortable space	On-going	Ongoing (before BAI implementation, and throughout the study, as needed)
Ensure that BAI training counts towards training credits for clinic counselors		• Clinic directors add BAI training as a training that counts towards clinical staff training credits	• Clinical staff are encouraged to participate in BAI trainings<br>• Demonstration that BAI is a priority to the clinic and clinic directors	Once	Once, before regional training
Central team, VAAC, and Provincial CDC leaders/Provincial Health Dept. conduct regional launching event and training	Clinic counselor	• VAAC, central team, and Provincial CDC leaders/Provincial Health Dept. prepare resources and organize a meeting with clinics to introduce the BAI<br>• Conducts a training on the BAI intervention, knowledge and skills for BAI delivery, the data collection system and other research procedures	• All stakeholders understand the nature of BAI intervention, design of an implementation study, timeline, budget, and human resources needed for the study at sites, so that they motivated to collaborate and implement BAI<br>• Leadership support and political will also motivate site staff to participate in BAI and implement BAI well<br>• Site staff understands BAI intervention and their responsibilities. They are also trained to have the necessary skills and knowledge to implement BAI well at their sites	Once	At the beginning of BAI implementation
Central team develops and delivers BAI training to clinicians		• Central team develops training materials<br>• Conducts a training on the BAI intervention, knowledge and skills for BAI delivery, the data collection system and other research procedures	• Site staff understands BAI intervention and their responsibilities. They are also trained to have the necessary skills and knowledge to implement BAI well at their sites	Once	At the beginning of BAI implementation
Develop graphics to assist measuring standard drinks for use in AUDIT		• Central team develops graphics for measuring standard drinks for AUDIT and distributes to sites<br>• Sites are trained in and implement use of graphics for measuring standard drinks as part of the AUDIT	• Site staff are better able to more consistently and accurately measure patients' alcohol use<br>• BAI is offered to all patients who need it	Once	Pre-BAI implementation

-Clinic leaders recognize and acknowledge that screening is being done<br>-Provide reminders to screen every time		• Periodic meetings with clinical staff to report on number of screenings conducted and to provide reminders about the importance of screening	• Clinic director support for screening will motivate site staff to screen patients consistently<br>• Site staff understands the importance of BAI screening and screen every patient	On-going	Throughout BAI delivery
-Clinic leaders recognize and acknowledge that referrals are being done<br>-Provide reminders to screen every time		• Periodic meetings with clinical staff to report on number of referrals conducted and to provide reminders about the importance of referring (when appropriate)	• Clinic director support for referrals will motivate site staff to refer patients consistently (as appropriate)<br>• Site staff understands the importance of referrals and refers patients, when indicated	On-going	Throughout BAI delivery
-Clinic leaders recognize and acknowledge that BAI sessions are being delivered<br>-Provide reminders to deliver and schedule the second session		• Periodic meetings with clinical staff to report on number of BAI sessions conducted and to provide reminders about the importance of BAI delivery when indicated	• Clinic director support for BAI session delivery will motivate site staff to deliver BAI sessions with consistency and fidelity<br>• Site staff understands the importance of BAI session delivery and deliver BAI sessions with fidelity	On-going	Throughout BAI delivery
-Clinic leaders recognize and acknowledge that booster sessions are being delivered<br>-Provide reminders to deliver and schedule the second session		• Periodic meetings with clinical staff to report on number of booster sessions conducted and to provide reminders about the importance of booster session delivery	• Clinic director support for BAI booster session will motivate site staff to deliver BAI booster sessions to every BAI participant with fidelity<br>• Site staff understands the importance of BAI booster sessions and deliver BAI booster sessions to every BAI participant with fidelity	On-going	Throughout BAI delivery
Bi-yearly regional facilitation calls	Facilitator	Facilitate group calls every 6 months for sites in the same arm	• Site staff knows how BAI is implemented at other sites and have the platform to share their knowledge and experience	Every 6 months	Every 6 months after enrollment
