# Supplementary material for: Scaling up a brief alcohol intervention to prevent HIV infection in Vietnam: a cluster randomized, implementation trial
Source: Implement Sci. 2024 Jun 12;19:40. doi: 10.1186/s13012-024-01368-6 (PMC11170841; doi:10.1186/s13012-024-01368-6)
Supplement: Supplementary file 2 — Additional file 2: Key implementation activities list. List of the key implementation activities for costing assessment. [file 13012_2024_1368_MOESM2_ESM.pdf]

Additional File 2: Key implementation activities

General Category (GC)	Activity Category (AC)	Description	Programmatic or Research	People involved
**Data Collection/Study Tools**	In-Depth Interview	This activity consists of in-depth interviews with health staff and PWHs.<br>**Inclusion:** 1) In-depth interviews with health staff in adaptive phase; 2) In-depth interviews with health staff at post training, at 12 and 24 months and 3) In-depth interviews with PWHs at 12 months after enrollment. These include developing plan, preparation of tools/guidelines and related procedures (contacting partners and logistics issues and in-depth interview), transcribing and analyzing the data with software and synthesis of findings.<br>**Exclusion:** Focus Group Discussion	50%: Programmatic<br>50%: Research	UNC VN staff, UNC Chapel hill
	Focus Group Discussion	This activity is a part of adaptive phase, which is organized with key stakeholders, including provincial CDCs, health staff at ART clinics.<br>**Inclusion:** Development of plan, preparation of guidelines and related documents, contacting partners and logistics issues for conducting FGDs, analyzing the data with software and synthesis of findings for inputs of next steps of BAI manual adaptation.<br>**Exclusion**: In-Depth Interview	70%: Programmatic<br>30%: Research	UNC VN staff, UNC Chapel hill
	Qualtrics survey	Qualtrics is an online survey tool (free) built to collect information to assess acceptability of the intervention and site context.<br>The central team sends to health worker participants the link of Qualtrics via email. They are completing the consent form at pre-training survey and surveys at pre and post training, 3-, 12- and 24-month post-implementation.<br>**Inclusion:** Qualtrics development, administration, and data analysis	30%: Programmatic<br>70%: Research	UNC VN

		**Exclusion:** PWID participant consent forms and surveys in Participant Tracking Database (PTD)		
	Quantitative assessment	Quantitative assessment with participants at follow-up rounds<br>**Inclusion:** These include preparation of tools, contact participants for arrangement of interviews, interview, and complete data in PTD system<br>**Exclusion:** Translation of questionnaires, qualitative data collections, Qualtrics surveys	100% research	UNC VN
	Development and revision of study materials	Study materials are developed and revised from the pre-implementation period to implementation period.<br>**Inclusion:**<br>1) All study procedures for site staffs and the central team involved in EBAI study's activities<br>2) Study tools (forms, guidelines and so on) applied for study sites in EBAI implementation<br>3) Other study materials (master plan/timeline, tracking file)<br>**Exclusion:** Intervention manual, PTD, protocol, fidelity forms and training materials, developing, and piloting questionnaires for assessment.	50%: Programmatic<br>50%: Research	UNC VN staff, UNC Chapel hill, HMU
	Development and Operation of Participant Tracking Database	The PTD is a software that collects research data and tracks the study progress of participants.<br>**Inclusion:** This activity includes developing requirements, testing PTD software, and operating it.<br>- Data management staff of UNC will develop requirements for the PTD software.<br>- The PTD software will be built by an outsourced IT company based on those requirements.<br>- UNC staff will check the operation of the software and find out any errors, then respond to the IT company to fix them until all the errors are resolved.<br>**Exclusion:** Budget development, contract development with the IT company	50%: Programmatic<br>50%: Research	UNC VN staff, TechLink (IT agency)

**Intervention & Implementation Strategy Activity**	Revision of Intervention Manual	Intervention manual is revised in adaptive phase in accordance with IDIs and FGDs results.<br>**Inclusion:** Effort for editing content, images, and formatting and translating<br>**Exclusion:** ToT/trainings on Intervention manual	100% Programmatic	UNC VN staff, UNC Chapel hill, a hired artist
	Clinic staff experience BAI	ART clinic staff at FAC+EBAI arm are delivered EBAI by counselors from central team before they deliver BAI for PWHs at their clinics.<br>**Inclusion:** 1) in-person BAI sessions, 2) Booster sessions via phone<br>These include development/revision of plan, completing encounter forms and required reports.<br>**Exclusion:** Trainings for 30 ART clinics or Training for counselors at clinics at FAC+EBAI arm after experience of BAI.	100% Programmatic	UNC VN, hire counselors
	Intervention mapping	Intervention Mapping (IM) is a multistep implementation process.<br>**Inclusion:** Development of tools/materials/implementation strategies for 30 ART clinics<br>**Exclusion:** Focus Group Discussion and In-depth interview	100% Programmatic	UNC VN, UNC Chapel hill, VAAC, HMU
	2-month group calls with ART clinics (by arm)	The central team will conduct regional group calls with ART clinics every 2 months to address barriers during the intervention implementation and share experiences as well as lessons learned.<br>**Inclusion:** Development of plan/guidelines/reporting forms, conduction of these calls and other related procedures.	100% Programmatic	UNC VN
	Technical assistance for clinic staff for delivering BAI to PWH participants	The central team will provide technical support for clinic staff at ART clinics regarding intervention delivery for PWH participants per requests.	100% Programmatic	UNC VN
**Meeting**	Team meetings for study progress	Team meetings are held for updating the study progress among the central team members (UNC, HMU and VAAC) or between the central team and stakeholders (provincial DOH and CDC, site leaders and staffs) or between Vietnam team and US team (not recorded hours meeting for US team)	Determined by meeting attendees	UNC, HMU, VAAC

		**Inclusion:** Meetings related to EBAI implementation including discussion on specific topic e.g., planning, revising study tools, BAI manual, technical issues<br>**Exclusion:** Meetings on the research issues; (Cost effectiveness, AEs/SAEs/SIs, DSMB, data collection and analysis)		
**Non-monitoring Site visit**	Initial site visit and assessment	The purpose of initial site visit and assessment is to 1) collect basic information about HIV/AIDS related services and processes at EBAI study sites; 2) introduce EBAI study to provincial CDC and sites; 3) discuss collaboration mechanism; 4) identify potential key staff to work with the study; 5) understand barriers influencing the intervention implementation and initially identify strategies to address them.<br>This helps to understand the context of sites and routine SOPs at sites at pre-implementation for site randomization.<br>**Inclusion:**<br>- Development of site assessment tools and guidelines for site visits<br>- Data collection/entry/cleaning<br>- Logistics issues and team meetings for sharing information on site visits and writing reports.<br>**Exclusion:** site randomization	90%: Programmatic<br>10%: Research	UNC VN, VAAC
	Interim visit	**Inclusion:** Development plan and conduction of interim visits<br>**Exclusion:** Planned monitoring visits and initial visits	60%: Programmatic<br>40%: Research	UNC VN, VAAC
**Administrative**<br>*(Includes: Approvals; Financial; Contracting)*	Authority approval for study implementation	These are required for implementing the EBAI study at 30 ART clinics of 10 provinces.<br>**Inclusion:** Preparing materials/documents and other required procedures for provincial authority approvals, including preparing add-on materials/reports/documents and other required procedures during study implementation<br>**Exclusion:** Submission to local IRB.	100% Programmatic	UNC VN, VAAC
	IRB approval	**Inclusion:** Preparing materials/documents and other required procedures for IRB approvals, including preparing add-on	100% research	UNC VN, VAAC, HMU

		materials/reports/documents and other required procedures during study implementation<br>**Exclusion:** Local authority approval		
	Development/ Review/ Revision of budget/ agreement/ general working plan/ financial and administrative procedures with partners/sites	**Inclusion:** This is required activity for implementing a study including development/review/revision of budget for all EBAI activities, annual working plan and related procedures with partners/sites, advance, reconciliation and payment for activities. It also involves in supporting and communicating on financial/administrative to implement EBAI study.<br>**Exclusion:** Development of detailed plans for specific activities	100% Programmatic	UNC VN, HMU
**Training**	Trainings for 30 ART clinics	**Inclusion:** 1) Initial training for 30 ART clinics, 2) Booster trainings and 3) Trainings for new staff.<br>These include development/revision of plan, training materials/tools, administrative/logistic assignments, training deployment, progress meetings and reports. Content of these training materials covers: study procedures/SOPs, BAI Counselors training, TLFB, GCP, SHP<br>**Exclusion:** EBAI delivery for health staff of 15 clinics (FAC+EBAI arm)	70%: Programmatic<br>30%: Research	UNC VN, VAAC, HMU
	Training for counselors at 15 ART clinic at FAC+EBAI arm	Training for counselors at FAC+EBAI arm to debrief about their experience before delivering BAI to PWH participants at their sites.	70%: Programmatic<br>30%: Research	UNC VN
	Trainings for trainers (TOT) for intervention manual and implementation science	These training include development/revision of plan, training materials/tools, administrative/logistic assignments, training deployment, meetings for preparation and reports. Content of these training materials will be solely for the intervention implementation. Thus, 100% time would be allocated for the programmatic component.	100% Programmatic	UNC VN, UNC Chapel hill
	Qualitative guides training	Trainings for interviewers and FGD facilitators on qualitative guides before conducting IDIs and FGDs in adaptive phase and implementation phase.	30% Programmatic<br>70% Research	UNC, HMU

**M&E**	M&E report	**Inclusion:** Review and QA, QC data to ensure the data quality. This include prepare QA, QC report, progress/key indicators report to study managers and PIs.<br>**Exclusion:** Monitoring visits and report, fidelity assessment	50% Programmatic; 50% Research	UNC VN, HMU
	Monitoring visit	**Inclusion:** Development plan and conduction of monitoring visits for documenting/reporting the study progress; updating any changes/amendment of study protocols/procedures; reviewing site data/source documents; checking required facility/equipment and providing feedback to improve quality of EBAI study implementation.<br>**Exclusion:** Listening counselling video tapes; Assessing quality of BAI counselling sessions for fidelity outcome	Estimated time for Programmatic and<br>Research purpose by performers	UNC VN, VAAC
	Fidelity assessment	**Inclusion:** Listening counselling video tapes; Assessing quality of BAI counselling sessions for fidelity outcome<br>**Exclusion:** Monitor BAI session at study sites to give health staff feedback to improve their skills as required.	100% Research	UNC VN
